# The Second Qatar Allergy and Immunology Conference: An Editorial

**DOI:** 10.5339/qmj.2023.sqac.1

**Published:** 2023-11-19

**Authors:** Maryam Ali AL-Nesf, Tayseer Ibrahim

**Affiliations:** ^1^Allergy and Immunology Division, Department of Medicine, Hamad Medical Corporation, Doha, Qatar, P.O. Box 3050 Email: Mariamali@hamad.qa

The 2^nd^ Qatar Allergy and Immunology Conference was organized by the Adult Allergy and Immunology Division of Hamad Medical Corporation (HMC) over two days as a hybrid conference on the 10^th^ and 11^th^ of February 2023 at ITQAN Clinical Simulation and Innovation Centre. The conference was preceded by two pre-conference workshops directed toward physicians and nurses on the 9^th^ of February, 2023. The workshops aimed to promote awareness about allergic and immunologic diseases among physicians and nurses.

The 2^nd^ Qatar Allergy and Immunology Conference represented a great learning platform for healthcare professionals and opportunities to update knowledge, present research, and meet professionals since allergy and immunology practice is a rapidly evolving science focusing on allergic and immunologic conditions and their advanced treatment options. The conference’s scientific program focused on the following topics: airway allergy and asthma, skin allergies, diagnosis and management of primary immunodeficiency diseases, and novel therapeutics for allergic diseases.

One of the significant achievements of this year’s conference is conducting a focused research symposium that provides a flagship platform for medical students and faculty scholars to present their research experiences and up-to-date health topics and to share their experiments and research findings with a broader community. It aimed to gather scientists and physicians from across Qatar and internationally with recent updates regarding the role of precision medicine in allergy and immunology science and practice.

HMC supported the event in partnership with the Qatar Allergy and Immunology Society (QAIS) (a chapter of the Qatar Medical Association). It was accredited for 21.25 CPD Category 1 as defined by the Ministry of Public Health’s Department of Healthcare Professions.

The conference hosted many expert speakers from Qatar, the Gulf region, and internationally. The total number of participants was 1082, comprised of physicians, nurses, and scientists. We received 34 abstracts, 32 were accepted for poster presentations, and the best six were selected for the oral presentation session.

Another achievement of our conference is advocating the Arabic language. One lecture and one oral abstract were presented in Arabic to demonstrate the importance and simplicity of using the Arabic language in medical education and science.

